# Valorisation of waste OSB into sustainable mycelium-based composites for insulation applications

**DOI:** 10.1038/s41598-026-52430-w

**Published:** 2026-05-13

**Authors:** Joni Wildman, Valeria Cascione, Daniel Henk, Pete Walker, Andrew Shea

**Affiliations:** 1 Department of Architecture and Civil Engineering, University of Bath, Bath, UK; 2 Institute of Sustainability and Climate Change, University of Bath, Bath, UK; 3 Milner Centre for Evolution, University of Bath, Bath, UK

**Keywords:** Engineering, Environmental sciences, Materials science

## Abstract

The need for the construction industry to reduce its contribution to landfill waste and greenhouse gas emissions is urgent, with engineered wood products such as oriented strand board (OSB) representing a complex and difficult-to-recycle waste stream. As the demand for construction materials increases, it is important to address end-of-life challenges for OSB. Mycelium-based composites (MBCs) offer a promising solution by repurposing waste materials into sustainable products. MBCs are a class of bio-based material made from the colonisation of fungal mycelium on organic substrates, often agricultural or paper waste. MBCs can provide an environmentally friendly end-of-life pathway for waste but also serve as effective insulation materials that can be sustainable alternatives to conventional insulation solutions. Here, MBCs are successfully produced using waste OSB and *Trametes versicolor* mycelium. The mycelium successfully colonised and bound the OSB substrate, forming that retained their shape after demoulding and drying. Thermal characterisation of the OSB-based MBCs showed a mean thermal conductivity of $\lambda = 0.0415 \pm 0.001$ W/m$\cdot$K, comparable to conventional insulation materials. Importantly, this approach opens up the possibility of including diverse materials as MBC substrates depending on local waste availability and waste challenges. A life cycle assessment (LCA) of the lab-scale production process was conducted to identify key areas of the production process that contribute to environmental impact, with drying being the most energy-intensive stage. The global warming potential (GWP) of OSB-based MBCs was found to be 0.1021 kg CO$_2$ eq per 1 kg of material, lower than conventional insulation materials such as EPS, XPS, and rockwool, and comparable to other MBCs made with alternative substrates. This work highlights the potential of valorising engineered wood waste, such as OSB, through the production of mycelium-based composites. By transforming construction waste into sustainable insulation materials, this study contributes to circular economy practices in the construction industry and supports efforts to reduce environmental impacts.

## Introduction

The construction industry is a major contributor to worsening climate crises, contributing approximately 36% of worldwide CO$_2$ emissions and generating 45-65% of the waste deposited in landfills^1,2^. Among the various waste streams associated with construction activities, wood waste is a significant issue, representing 20-30% of construction and demolition (C&D) waste and accounting for 10% of total landfill waste^3^. Of this waste wood, treated wood products, such as oriented strand board (OSB), are considered complex waste streams due to the resin content^4–6^. OSB is a widely used engineered wood panel board product, formed from compressed layers of wood strands (flakes) in specific orientations bonded together by adhesives under high temperature and pressure. The most common uses are as sheathing in walls, flooring, and roof decking^7^. Common resins used in OSB include urea-formaldehyde, isocyanate-based glue (pMDI), melamine-urea-formaldehyde, and phenol formaldehyde^8^. The synthetic resin content in OSB raises environmental problems due to their impact on incineration and landfill disposal, a consequence of resins limiting the material’s biodegradability^9^. Guidance on wood waste disposal states that landfill and incineration should be of the last resort, with recycling being preferable, however, there are no widely established recycling pathways for OSB^10^. To address these concerns, bio-based adhesives derived from lignin, starch, and tannins are being explored as sustainable alternatives. These adhesives offer the potential to reduce environmental impacts and improve OSB’s biodegradability. However, they are still in the developmental stage, with challenges such as poor water resistance and lower bonding strength compared to conventional adhesives. Further research is needed before bio-based adhesives can be widely adopted in OSB production^11–13^.

The incineration of OSB specifically is associated with environmental and health concerns. The combustion process releases volatile organic compounds (VOCs), including aldehydes and formaldehyde-based compounds, which vary depending on the type of resin used in the OSB. VOCs are recognised by the Environmental Protection Agency (EPA) and the National Institute for Occupational Safety and Health (NIOSH) as hazardous to human health and air quality, with some compounds classified as carcinogens^14,15^. Furthermore, incineration emits harmful pollutants such as carbon monoxide (CO) and nitrogen oxides (NOx), which also pose serious health risks as well as contributing to environmental issues such as smog formation and acid rain^16^. Greenhouse gas (GHG) emissions from burning OSB further exacerbate the environmental impact. The process releases stored biogenic carbon, sequestered by the wood, back into the atmosphere; this can be intensified by the resin content, resulting in a higher GHG footprint compared to other wood-based products^17^.

The environmental challenges associated with OSB landfill disposal are also significant. Unlike untreated wood, the synthetic resins and adhesives in OSB impede its decomposition, leading to a prolonged release of GHGs under anaerobic landfill conditions. As OSB decomposes, potent GHGs (methane and carbon dioxide) are emitted. Methane emissions are concerning as methane has a higher global warming potential than carbon dioxide^9,18^.

The composition of OSB, including lignin and synthetic adhesives, complicates its environmental footprint in landfills. Lignin, being highly resistant to microbial degradation, slows the decomposition process and results in the persistence of biogenic carbon in landfill environments^19^. Furthermore, the OSB adhesives contribute to pollutants in landfill leachate, such as zinc-borate, which necessitate leachate treatment systems to prevent contamination of surrounding soil and groundwater^20,21^.

In addition to the direct environmental impacts, the slow decomposition of waste in landfills increases the demand for landfill space. This delays the availability of land for alternative uses, such as agriculture, residential development, or natural restoration^9,22^.

Despite the environmental drawbacks associated with incinerating or landfilling OSB waste, recycling options for the material remain limited^23^. The construction industry is expanding globally^24^, with the increasing demand for engineered wood products leading to a growing volume of OSB offcuts and post-consumer waste^25^. Consequently, there is an urgent need for more innovative end-of-life uses for OSB and similar engineered wood products. The valorisation of OSB waste into mycelium-based composite insulation materials offers an sustainable solution, transforming waste into valuable bio-based insulation materials. This approach supports circular practices in the construction industry by providing a sustainable end-of-life for OSB waste, and additionally provides an alternative to unsustainable, energy-intensive petrochemical-based insulation materials^26^.

Mycelium-based composites (MBCs) are a class of bio-based material formed from the growth of fungal mycelium on organic substrates^27^. Mycelium is the vegetative structure of fungi, consisting of a network of branching filaments called hyphae. The fungal mycelium digests organic material by breaking down polymers such as lignin and cellulose. Fungi play an important role in natural material decomposition and recycling, using enzymes that are secreted directly on to the subtrate to do so^28^. This digestion process, which occurs as the fungus colonises the material, forms a natural adhesive which binds the substrate together into a matrix. The fully colonised substrate is then dried at elevated temperatures (40-50 $^\circ$C) to inactivate the living fungus, resulting in a non-living bio-composite^29^.

MBCs can exhibit favourable thermal, acoustic, and fire-resistant properties^30–32^. They also have environmental benefits such as being biodegradable, capable of utilising waste streams, and being associated with a low embodied carbon^27,33,34^. In contrast, conventional insulation materials such as expanded polystyrene (EPS) and extruded polystyrene (XPS) are non-biodegradable, energy-intensive to produce, and rely on finite resources^35^. The transition away from these unsustainable materials is necessary in the context of the goal of a more circular construction industry. MBCs offer potential to reduce both operational and embodied carbon emissions from buildings; they can reduce the need for landfill and incineration, reduce material production carbon emissions, and reduce energy demands for the heating and cooling of buildings.

As the substrate, MBCs commonly use waste materials from agriculture, such as straw and hemp, as well as by-products from the wood industry, including wood chips and cork, and paper industry waste^36^. While these are often classified as waste materials, they retain value in other end-of-life applications such as animal feed, textiles, and pulping^37^. Fungi are known to produce a wide range of extracellular enzymes capable of degrading complex lignocellulosic compounds such as cellulose and lignin. This degradative capability enables many fungi to colonise and grow on lignocellulosic substrates^38–40^. This presents an opportunity to repurpose difficult and lower-value waste streams from the construction industry, such as engineered wood (e.g. OSB) waste.

The aims and objectives of this study are as follows:

- To investigate the feasibility of valorising OSB as a substrate for the production of mycelium-based composites.
- To evaluate the thermal performance and effectiveness of OSB-MBCs as insulation materials.
- To conduct a lab-scale life cycle assessment (LCA) of OSB-MBCs to provide insights into their environmental impacts and compare these impacts with other MBCs and conventional insulation materials.

This study demonstrates that fungi can grow on and bind adhesive-containing engineered wood waste. This establishes the feasibility of using waste OSB as a substrate for producing MBCs with low thermal conductivity, suitable for sustainable insulation applications. OSB-based MBCs were successfully produced and their thermal performance characterised, and a LCA was conducted to evaluate the environmental impacts of their lab-scale production. The development of OSB-based MBCs presents a dual opportunity: offering a sustainable alternative to conventional insulation materials while valorising waste from the construction industry, therefore making progress towards a more circular construction industry and lowering carbon emissions in the built environment.

## Methods

The methodology used in this study involved the valorisation of waste oriented strand board (OSB) by using this as a substrate for producing mycelium-based composites. The sourcing and processing of the OSB is described, as well as the mycelium-based composite production process. The thermal conductivity of the produced MBCs was measured using a steady state methodology and the density of the materials measured. Additionally, a life cycle assessment of the lab-scale production of the MBCs was conducted, covering stages A1-A3 (cradle-to-gate), and the contribution to the environmental impacts from the different life cycle stages as well as MBC production stages was analysed. The contribution to environmental impacts from electricity and materials was also calculated.

### Mycelium-composite production

#### OSB substrate preparation

Waste off-cuts of oriented strand board (OSB) were sourced from the University of Bath department of Architecture and Civil Engineering wood-work laboratories. The OSB used in this study was a commercial structural panel manufactured by Kronospan and bonded using polymeric diphenylmethane diisocyanate (pMDI) resin, which typically represents approximately 2-4% of the total board mass^41^. Such isocyanate-based adhesives are commonly used in OSB manufacturing due to their moisture resistance and strong bonding performance^42^. The waste OSB, with density 600 kg/m$^3$ was processed on site with a wood chipper. The chipped OSB was passed through a 30 mm sieve to remove oversized pieces. The resulting particles were irregular flakes typically measuring approximately 10-30 mm $\times$ 10-20 mm, although particle size distribution was not formally characterised.

The processed OSB was soaked in water for 24 h. Subsequently the OSB was drained and mixed with plain flour (1:10 ratio flour to OSB) to serve as an additional nutrition source for more rapid fungal growth. This mix was packed in to an autoclavable bag before being sterilised in an autoclave.

#### Fungal culture preparation

A specimen of *Trametes versicolor* was identified in Bath, UK, based on observable characteristics of it’s fruiting body. The identity of the species was verified based on sequencing of the ITS4 region. The ITS sequence generated in this study has been deposited in GenBank under accession number PZ140950. The specimen was cloned with the mycelium subsequently being propagated, maintained, and stored on malt extract agar with yeast extract (MEYA), a commonly used nutrient-rich medium for maintaining basidiomycete cultures and supporting mycelial growth^43^.

Mycelium growing on MEYA was then used to inoculate hydrated and sterilised brown rice. The fungi was allowed to colonise the rice for 5 days in an incubator. The fungi was incubated at 23 $^\circ$C, which lies within the typical temperature range reported to support mycelial growth of many basidiomycete fungi, including *Trametes versicolor*, under laboratory culture conditions^43^. The grain colonised by mycelium was subsequently used as grain spawn to form the composites.

#### Composite production

**Fig. 1:**
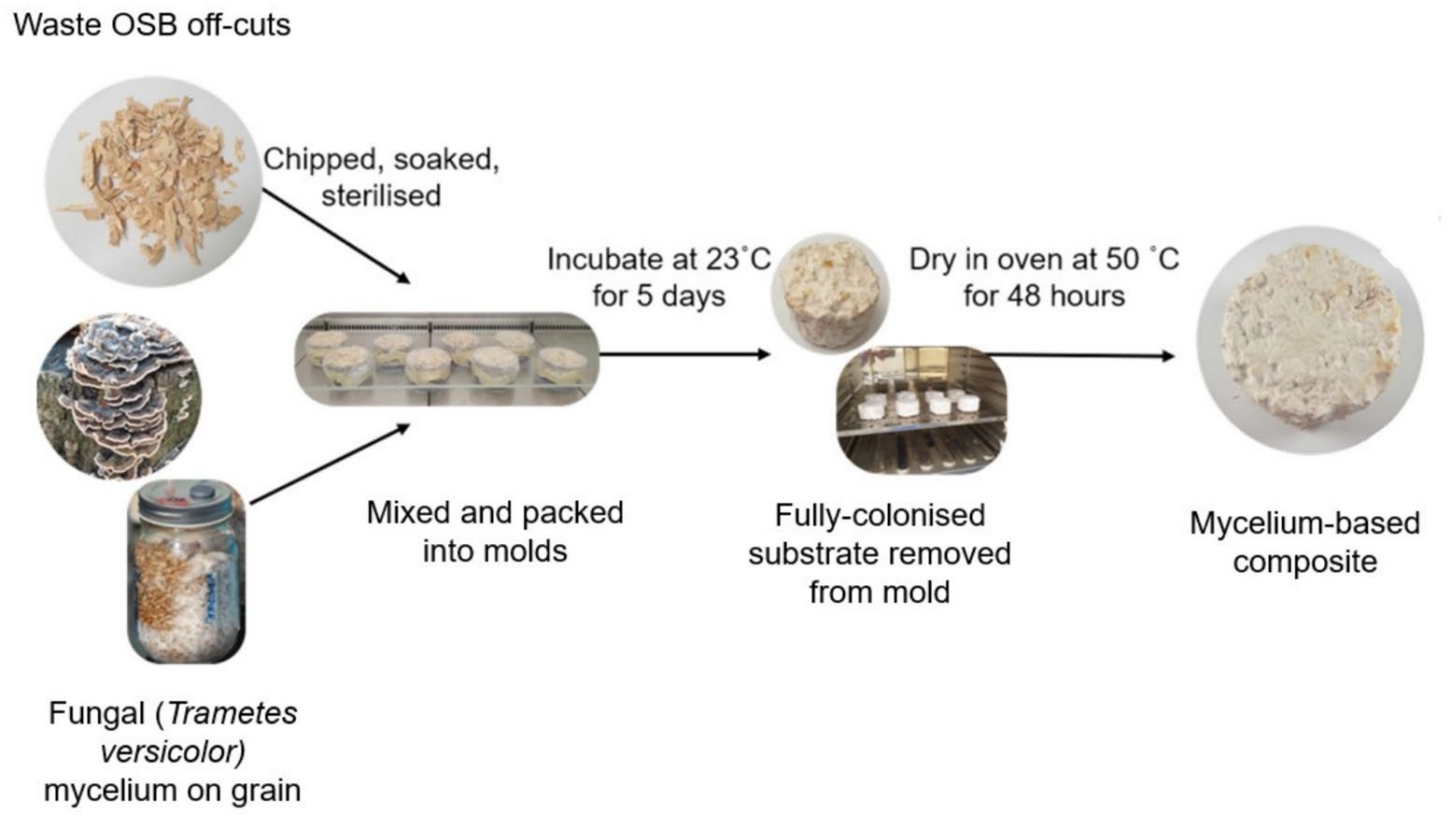
Steps in the mycelium-based composite production process. Prepared and sterilised substrate (OSB chips) are mixed with fungal grain spawn and packed into a mould. The moulds are covered and placed in an incubator at 23 $^{\circ }$C for 5 days to allow the fungal mycelium to colonise the material. Once fully colonised, the composite is dried in an oven at 50 $^{\circ }$C for 48 h; this step inactivates the fungus to leave an inert bio-based material.

Figure 1 shows a diagram of the composite production process. In a biosafety cabinet the substrate and grain spawn were combined in a ratio of 10:1 with the grain spawn broken up into mycelium-colonised individual grains. A large bowl and spoon, cleaned with 70% isopropanol, were used to mix the spawn and substrate.

The mixture was then packed in to cylindrical moulds (height 50 mm, diameter 80 mm) cleaned with 70% isopropanol. Once the moulds were filled they were covered with a sheet of parafilm secured with tape to allow for air exchange whilst minimising contamination during incubation.

The specimens were then incubated at 23 $^\circ$C for 5 days. The specimens were then removed from the moulds and weighed. They were then dried in an oven at 50 $^\circ$C for 48 h. The specimens were weighed after 40 h and after 48 h to ensure that no further mass was lost upon 8 h additional drying thus the specimens were not retaining moisture. Eight biological repeats were made.

### Thermal characterisation

The thermal conductivity of each specimen was determined using a Heat Flow Meter according to ASTM-C518^44^. Five thermal analysis tests were performed on each of the eight specimen (40 tests in total) using a Thermtest HFM-25. Specimen density ($\rho$) was recorded prior to thermal characterisation ($\rho = 258 \pm 3$ kg/m$^3$). Each specimen was stored in the laboratory at room conditions for 1 week (approx. 20 $^{\circ }$C and 50% RH) prior to testing. The test was conducted at a mean temperature of 20 $^{\circ }$C with a 20 $^{\circ }$C temperature difference between the plates.

### LCA

#### Goal and scope

The assessment was conducted to quantify the impacts of valorising OSB waste by using it as a substrate for the production of MBCs (of which can be used as insulation materials amongst other uses such as packaging). The goal of the study is to assess the environmental impacts of utilising waste OSB as a substrate for MBC production, and identify areas of improvement. To achieve this goal, the declared unit (DU) was considered: 1 kg of MBC produced. This equated to the use of 0.91 kg of waste OSB. The choice of mass for the DU, as opposed to a DU (or functional unit) that considered thermal performance, was because the use case for the produced MBC could have alternative uses to thermal insulation, such as acoustic insulation or packaging^45^. The quantities and processes required to make this DU are included in the supplementary information.

**Fig. 2:**
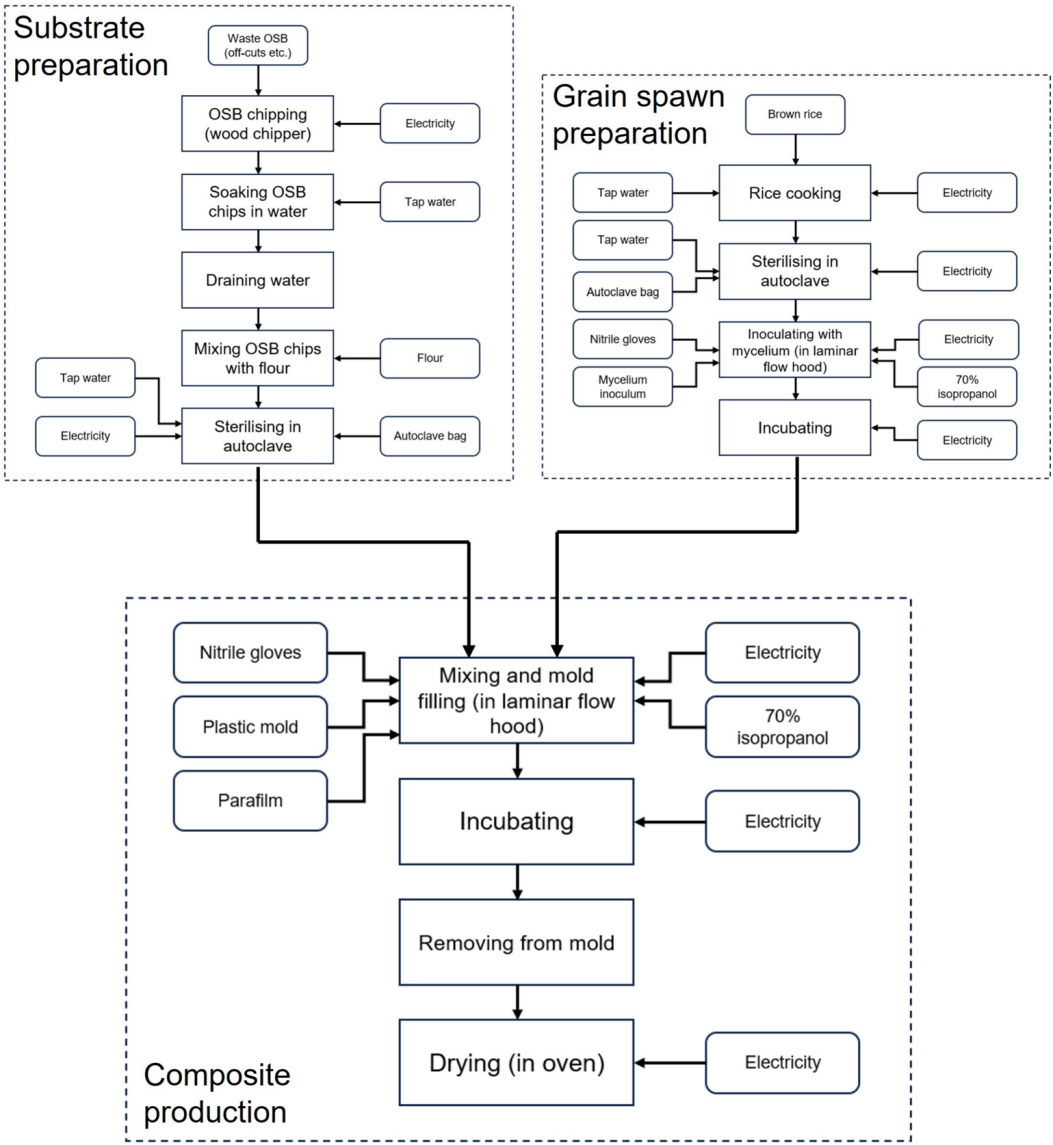
Stages in the OSB-MBC production process modelled in the LCA in this study. The diagram illustrates the life cycle assessment (LCA) steps, including the flows and processes included for the OSB-MBC production. The substrate is prepared by chipping, soaking, and sterilisation. The grain spawn in prepared by inoculating sterilised rice with fungal mycelium. The substrate and grain spawn and then combined to form the composite by packing the mixture in to moulds, incubating, and drying the colonised material.

The LCA was completed in alignment with EN 15804:2012+A2:2019^46^. The cradle-to-gate (A1-A3) impacts of lab-scale production of OSB-MBCs were considered, with the system boundaries depicted in Fig. 2. OpenLCA 2.3.0 was used for the assessment. The Life Cycle Impact Assessment (LCIA) and LCA studied the potential impacts on human health, natural environment, and natural resources; the global warming potential impacts were chosen as the focus in this paper due to its global relevance and the availability of robust characterisation models. LCIA was done in accordance with EN 15804:2019, using the EuGeos impact assessment method.

The buckets used to soak substrates were not included in the LCA as they can be reused without limit. The production of capital goods (e.g. autoclave, laminar flow hood) was also not included as previous studies have shown their influence on the LCA of construction materials is not significant^47^. The production of mycelium inoculant (mycelium stored on agar) is also not included as only a small amount of mycelium is required to produce a comparatively large amount of grain spawn, which can inoculate a comparatively large volume of substrate because of the expansion of mycelium when it grows on a substrate.

#### Inventory

Data collection were mainly conducted through laboratory experiments, supplemented by data from the Ecoinvent V3.6 database^48^, and, where experimental data were unavailable, relevant literature sources^49–52^. Details of the life cycle inventory (LCI), including quantities, data sources, and assumptions are provided in the supplementary information (SI). The Ecoinvent V3.6 database served as the background database, and OpenLCA^53^ was used for the LCA analysis. Electricity consumption was modelled using electricity supply datasets from the Ecoinvent database representing the United Kingdom grid mix (GB).

Biogenic carbon accounting follows the EN 16485:2023 guidelines, which provide a framework for assessing biogenic carbon flows in wood-based products^54^. The biogenic carbon stored in the OSB was calculated following the standard EN 16449:2014^55^, according to the formula: $P_{CO_2} = \frac{44}{12} \times cf \times \frac{m}{1 + \frac{w}{100}}$, where $P_{CO_2}$ is the biogenic carbon storage in the OSB (kg CO$_2$ eq), *cf* is the carbon fractional content of the OSB (0.463 for OSB^56^), *m* is the mass of the OSB, and *w* is the moisture content of the OSB (7%^57^)^17^.

#### Sensitivity analysis

A one-at-a-time sensitivity analysis was conducted to assess the influence of uncertainty in key foreground parameters on the global warming potential (GWP) results. The electricity consumption associated with the three most energy-intensive stages of the laboratory production process (drying, incubation, and substrate sterilisation) was varied individually by ± 10 % relative to the baseline inventory values. For each parameter variation, the life cycle model was recalculated in openLCA while holding all other inputs constant. The resulting GWP values were obtained from the impact assessment and compared to the baseline result to quantify the relative change in total GWP.

## Results

### Composites

**Fig. 3:**
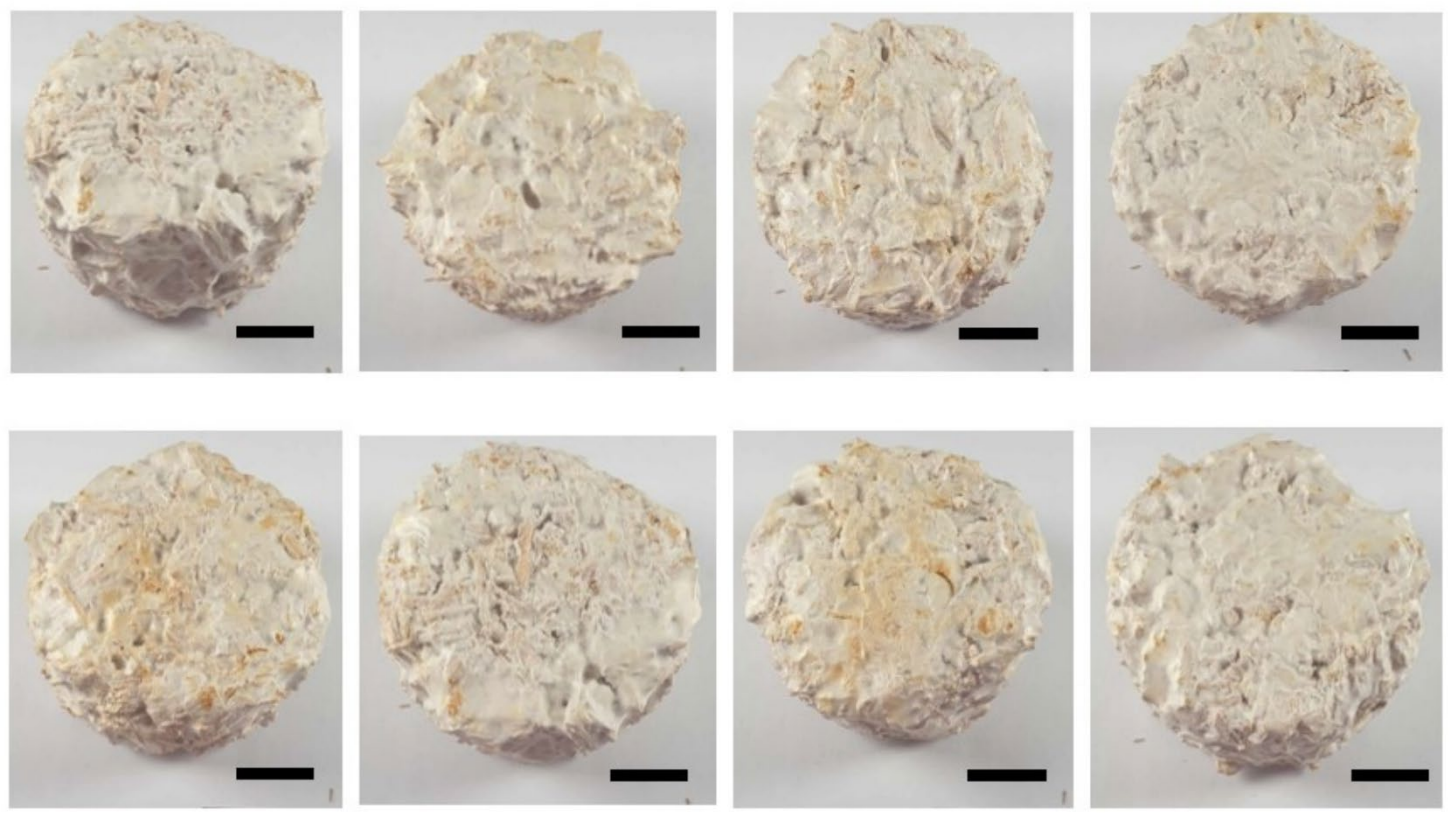
Macroimages of the OSB-MBC specimens produced in the study, showing the structural integrity and uniform colonisation of the OSB substrate by *Trametes versicolor* mycelium. Scale bar = 20 mm.

Figure 3 shows the OSB-MBCs produced for this study. *Trametes versicolor* successfully colonised the OSB substrate and produced cohesive composite specimens that could be demoulded and handled without disintegration. Eight replica specimens were produced with good consistency between specimen. Colonisation of the mycelium on the OSB substrate was homogeneous and complete. This demonstrates the feasibility of MBC production using OSB substrates and the ability of fungi to grow on treated wood products.

### Thermal performance

**Fig. 4:**
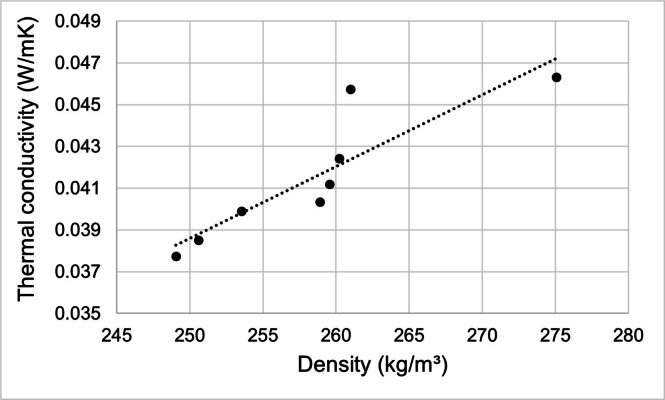
Plot showing the thermal conductivity of the OSB-MBC specimens (as measured using the Heat Flow Meter method) as a function of their density. The average thermal conductivity is $\lambda = 0.0415 \pm 0.001$ W/m$\cdot$K and average density is $\rho = 258 \pm 3$ kg/m$^3$. A statistically significant large positive correlation ($r = 0.886, p = 0.001$) is observed.

The average values were measured as $\lambda = 0.0415 \pm 0.001$ W/m$\cdot$K and $\rho = 258 \pm 3$ kg/m$^3$. Figure 4 shows the thermal conductivity ($\lambda$) of the specimens plotted as a function of their density ($\rho$). A Pearson’s correlation test was conducted to assess the relationship between $\lambda$ and $\rho$. $\lambda$ and $\rho$ showed a statistically significant large positive correlation ($r = 0.886, p = 0.001$) indicating that $\lambda$ increases as $\rho$ increases.

### LCIA

Table 1 shows the environmental impact of the production of 1 kg of OSB-MBC following the laboratory method detailed in the method section. The total global warming potential (GWP) was calculated as 0.1021 kg CO$_2$ eq.

Table 2 compares the total GWP of 1 kg of the lab-scale OSB-MBC with results from previous LCAs (obtained from the literature) of mycelium-based composites (MBCs) produced with different substrates, as well as bio-based and conventional insulation materials. Additionally, the table includes a scenario where the transport of OSB waste (assumed 100 km) is included in the analysis. To produce the MBCs in this study, the OSB waste was sourced on-site, but this additional scenario was considered to assess the feasibility of using OSB waste as a substrate in cases where it must be transported to the production facility prior to processing.

**Fig. 5:**
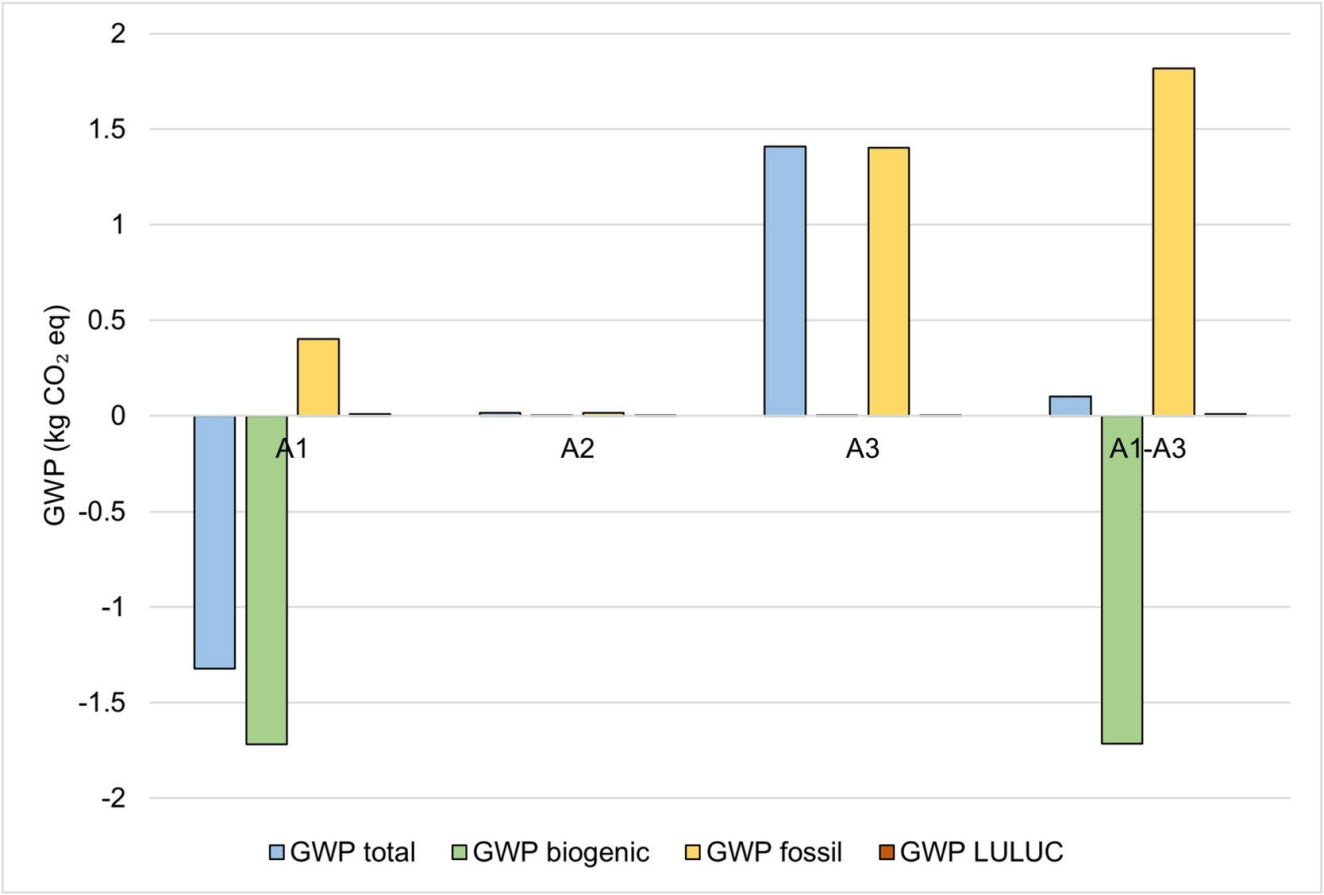
Graph showing the contribution to GWP (total, biogenic, fossil, and LULUC) of 1 kg of OSB-MBC, with OSB located on-site, from different stages (A1-A3) of the material life cycle.

Figure 5 illustrates the contributions to the GWP indicators (total, biogenic, fossil, and land-use-and-land-use-change (LULUC)) across the A1-A3 stages of the OSB-MBC life cycle, encompassing material production, material transport, and MBC manufacturing. The total GWP was highest in stage A3, indicating the production process as an important area for reducing overall GWP, and therefore elements of the production process (e.g. incubation time, sterilisation method) as targets for improvement.

Stage A1 exhibited the greatest negative GWP of -1.72 kg CO$_2$ eq due to carbon sequestration associated with the biogenic materials, specifically the wood-based OSB, as well as the grain and flour. Stage A3 showed the highest contribution to GWP fossil, due to the energy demands of the incubator and the oven for drying the specimens, both of which require prolonged use (5 days and 48 h respectively). Unlike in the A1 stage, the GWP fossil impact is not mitigated by any biogenic carbon storage and therefore the total GWP in the A3 is net positive. Although the GWP total is negative in the A1 stage, there is still an associated GWP fossil impact associated with material processing.

**Fig. 6:**
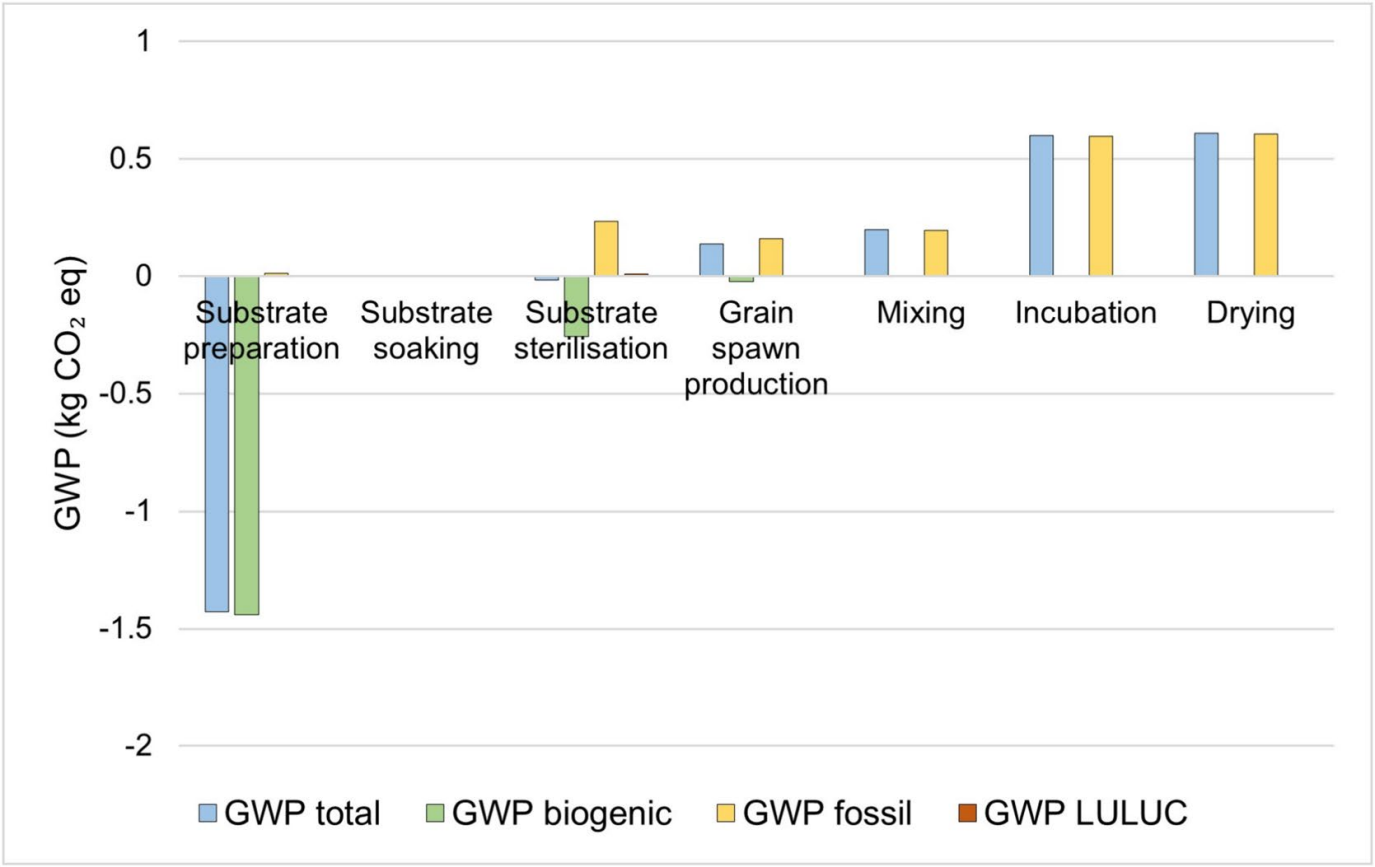
Contribution to GWP (total, biogenic, fossil, and LULUC) of 1 kg of OSB-MBC from different production stages of the material (substrate preparation, substrate soaking, substrate sterilisation, grain spawn preparation, mixing, incubating, and drying). The stage with the highest total GWP was the drying, accounting for 33.6% of total GWP.

Breaking down stages A1-A3 into specific steps within the production process, including the production and transport of materials associated with each stage, provided further insight into the most impactful aspects of the process. Figure 6 illustrates the contributions to GWP (total, biogenic, fossil, and LULUC) for the key steps involved in this MBC production. The drying stage was found to have the highest total GWP, primarily due to the significant electricity consumption required to dry the material in an oven over a 48 h period. The drying and incubation stages contributed 33.63% and 33.05% to GWP fossil, respectively, representing the largest net contributions to the total GWP. Unlike the substrate preparation, substrate sterilisation, and grain spawn production stages, where OSB, flour, and grain result in negative GWP biogenic, there is no biogenic carbon storage associated with the incubation, mixing, or drying stage. Consequently, the total GWP is largely driven by GWP fossil emissions during the mixing, incubation, and drying stages. Additionally, the influence of GWP LULUC on GWP total is minimal across all stages.

**Fig. 7:**
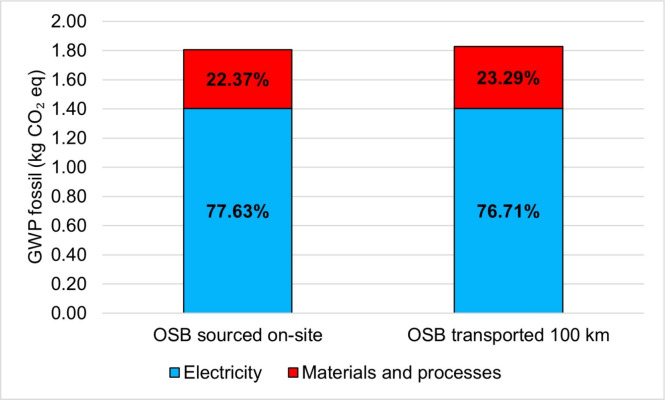
Contribution to total GWP of 1 kg of OSB-MBC from electricity vs materials and processes for two cases: with waste OSB located on-site, and with waste OSB transported 100 km before processing. Electricity remains the dominant contributor in both scenarios.

To better understand the contributions of different elements of production to the overall impacts, the percentage contributions of electricity versus materials and processes were calculated. This analysis was performed for two scenarios: one where OSB waste is sourced on-site (as was the case in this study) and one where OSB waste is transported 100 km to the site before processing. The results are presented in Fig. 7. Transporting the OSB 100 km increased total GWP from 0.1021 kg CO$_2$ eq to 0.1237 kg CO$_2$ eq.

When OSB waste was sourced on-site, electricity accounted for 77.6% of the GWP fossil, while in the scenario with 100 km of transport, electricity contributed 76.7%. In both cases, electricity remained the dominant contributor to GWP fossil.

Table 3 shows the results of the sensitivity analysis examining the influence of variations in electricity consumption during key production stages on the total GWP. The sensitivity analysis showed that the GWP results were strongly influenced by electricity consumption associated with the drying and incubation stages. A ± 10% variation in drying electricity resulted in changes of approximately ± 49% in total GWP, with the impact decreasing from 0.124 kg CO$_2$ eq to 0.0626 kg CO$_2$ eq when electricity demand was reduced and increasing to 0.185 kg CO$_2$ eq when electricity demand was increased. A similar pattern was observed for incubation electricity, where a ± 10% variation resulted in changes of approximately ± 48% in total GWP. Although these percentage increases are large, this may partly reflect the relatively low baseline total GWP.

In contrast, total GWP was less sensitive to the substrate sterilisation electricity input. A ± 10% variation in sterilisation electricity resulted in changes of approximately 6% in total GWP, with GWP values ranging from 0.116 to 0.131 kg CO$_2$ eq.

These findings indicate that the environmental impacts of the laboratory-scale OSB-MBC production process are sensitive to electricity consumption associated with incubation and drying, which were previously identified as the dominant contributors to the total GWP. Improvements in energy efficiency or reductions in electricity demand during these stages would therefore likely result in substantial reductions in overall environmental impact.

The electricity consumption in the LCA was modelled using the Great Britain (GB) electricity grid mix. The environmental impact associated with electricity use depends on the regional electricity generation mix, which varies according to the relative contributions of renewable and fossil-based energy sources. However, the electricity grid in a given location is often beyond the control of producers, limiting opportunities to choose lower-carbon energy sources. Energy efficiency improvements in production processes can be made however, such as optimising drying times, adopting alternative sterilisation methods, and reducing incubation temperatures.

**Table 1:** Environmental impact indicators for the conducted LCA of 1 kg of OSB-MBC produced at the laboratory scale using 0.91 kg of waste OSB, in accordance with EN-15804. Acronyms: GWP-biogenic (climate change, biogenic), GWP-fossil (climate change, fossil), GWP-LULUC (climate change, land use and land use change), GWP-total (climate change, total), ODP (ozone depletion), AP (acidification), EPf (eutrophication, freshwater), EPm (eutrophication, marine), EPt (eutrophication, terrestrial), POCP (photochemical ozone formation), ADPE (resource use, minerals and metals), and ADPF (resource use, fossils).

Impact category	Reference unit	Value
GWP- Total	kg CO$_2$ eq	0.1021
GWP- Biogenic	kg CO$_2$ eq	-1.7148
GWP- Fossil	kg CO$_2$ eq	1.8063
GWP- LULUC	kg CO$_2$ eq	0.0100
ODP	kg CFC11 eq	1.65E-07
AP	molc H+ eq	0.0090
EP f	kg P eq	5.18E-04
EP m	kg N eq	0.0033
EP t	molc N eq	0.0249
POCP	kg NMVOC eq	0.0043
ADPE	kg Sb-Eq	1.12E-05
ADPF	MJ	32.11
WDP	m$^3$	158.11

**Table 2:** Comparison of global warming potential (GWP) results for life cycle stages A1-A3 reported per 1 kg of material and per functional unit corresponding to 1 m$^2$ of insulation providing a thermal resistance of R = 1 m$^2$K/W. Results include different mycelium-based composites (MBCs) from published LCAs as well as selected bio-based and conventional insulation materials based on published LCAs and Environmental Product Declarations (EN 15804+A2).

Material	GWP total (kg CO$_2$ eq) per 1 kg	Density (kg/m$^3$)	Thermal conductivity (W/m$\cdot$K)	GWP total (kg CO$_2$ eq) per 1 m$^2$ thickness required to provide R = 1 m$^2$K/W	Ref.
Sawdust-based MBC	0.053	212	0.05	0.56	^58^
Hemp-based MBC	0.37				^59^
Bamboo-based MBC (”MycoBamboo”)	0.65	229	0.05	7.43	^60^
Hemp-based MBC	1.38				^61^
Cellulose and Rapeseed-straw-based MBC	-0.24	163	0.04	-1.58	^62^
(On-site sourced) Waste-OSB-based MBC	0.10	258	0.042	1.09	This study
(Transported 100km) Waste-OSB-based MBC	0.12	258	0.042	1.32	This study
Wood fibre insulation	-0.32	55	0.037	-0.64	^63^
Straw insulation	0.55	100	0.052	2.88	^64^
Rockwool	1.40	77	0.035	3.77	^65^
Extruded polystyrene (XPS)	98	30	0.034	100	^66^
Expanded polystyrene (EPS)	126	15	0.040	75	^66^
Polyurethane (PUR)	124	35	0.023	100	^66^

**Table 3:** Sensitivity analysis results showing the effect of ±10% variation in electricity consumption during drying, incubation, and sterilisation stages on the total global warming potential (GWP) of OSB-MBC production. Baseline electricity values are varied while all other parameters remain constant, and the resulting changes in GWP are reported relative to the baseline result.

Input	Baseline value (MJ)	-10% value (MJ)	+10% value (MJ)	GWP baseline (kg CO$_2$ eq)	GWP -10% (kg CO$_2$ eq)	GWP +10% (kg CO$_2$ eq)	Change -10% (%)	Change +10% (%)
Drying electricity	5.8	5.2	6.4	0.124	0.0625	0.185	49.52	49.19
Incubation electricity	5.7	5.13	6.27	0.124	0.0637	0.184	48.63	48.39
Substrate sterilisation electricity	0.72	0.65	0.80	0.124	0.116	0.131	6.45	5.65

## Discussion

This study demonstrates that fungi, specifically *Trametes versicolor* can effectively colonise and digest waste-OSB to produce a sustainable mycelium-based composite with low thermal conductivity. Although OSB contains synthetic adhesives that can complicate recycling, the successful colonisation observed in this study suggests that the presence of these binders did not prevent mycelial growth on the substrate. The resulting MBCs formed cohesive composite structures with a uniform appearance and were produced within a timeframe comparable to MBCs based on agricultural substrates, demonstrating the ability of fungi to colonise and bind engineered wood waste substrates. Further work involving chemical or microstructural analysis would be required to determine whether any modification or degradation of the adhesive phase occurs during colonisation.

Heat Flow Meter thermal conductivity measured a mean thermal conductivity of $\lambda = 0.0415 \pm 0.001$ W/m$\cdot$K, and mean density was measured as $\rho = 258 \pm 3$ kg/m$^3$. This is comparable to thermal measurements made of MBCs, which have measured thermal conductivity ranging from 0.026 W/m$\cdot$K to 0.18 W/m$\cdot$K^30^. Furthermore, this is comparable to conventional insulation solutions (XPS $\lambda = 0.033$ W/m$\cdot$K, EPS $\lambda = 0.036$ W/m$\cdot$K, rockwool $\lambda = 0.035$ W/m$\cdot$K)^65,67,68^.

Thermal conductivity displayed a statistically significant positive correlation with density. One possible explanation is that higher-density specimens may contain a greater proportion of solid material relative to air-filled voids, which can increase conductive heat transfer pathways through the solid phase. However, the present study did not include direct measurements of porosity or microstructural characterisation of the composites. A positive correlation has been observed in different bio-based insulation materials, however other studies report different trends within different density ranges and therefore care is to be taken when extrapolating^69,70^ Further optimisation of $\lambda$ could focus on density reduction, possibly by reducing the amount of grain spawn used during the composite mixing stage, or packing the OSB chips more lightly. This assessment of thermal performance is promising, and further characterisation of durability, hygroscopic properties and fire safety would help to assess the potential for the use MBCs made with waste engineered wood within in the built environment.

In this study, the LCA was conducted using a declared unit (DU) based on mass (1 kg of material produced) in order to reflect the potential versatility of the composite for applications beyond thermal insulation, such as acoustic insulation or packaging^45,71^. When comparing this DU with equivalent mass-based results reported for conventional insulation materials and previously published LCAs of MBCs, the OSB-MBC produced in this study shows a lower total GWP than conventional insulation materials and falls within the range reported for other MBCs. To enable comparison based on insulation performance, the DU was also scaled to a functional unit (FU) corresponding to the mass of material required to provide 1 m$^2$ of insulation with a thermal resistance of R = 1 m$^2$K/W. Under this FU, OSB-MBCs exhibit substantially lower GWP than conventional insulation materials such as EPS, XPS, and polyurethane (PUR), despite these materials having lower densities and higher thermal performance per unit thickness. Compared with other bio-based insulation materials, the GWP of OSB-MBCs is broadly comparable to that of straw insulation and higher than wood fibre insulation, while remaining within the range reported for other MBC systems in the literature. In addition to their environmental performance, OSB-MBCs offer the advantage of valorising a challenging engineered wood waste stream, providing both material recovery and insulation functionality. This further highlights the environmental benefits of mycelium-based composites in reducing carbon emissions in the construction industry. At the lab scale, the MBC production process exhibits variability; despite this, it is reassuring that across the reviewed MBC LCA studies, the total GWP consistently remains lower than that of conventional insulation materials.

The LCA focused on lab-scale production, with environmental impacts likely to change with scale-up. Scale-up of mycelium composite production may reduce the relative energy demand of several processing steps through improved process efficiency and equipment utilisation^58^. For example, industrial production could employ bulk incubation systems and continuous or batch drying processes with improved heat recovery. This initial assessment can be instructive in identifying key production stages for potential emission reductions as the material is further developed. The drying stage contributed 33.6% to GWP-total, the highest contributing stage to this category. To reduce emissions as the process is scaled, energy-efficient drying methods should be considered. For example, after the initial heating phase to inactivate the fungus, low-energy approaches such as desiccators could be used to dry the specimens. Volk et al. conducted an LCA of hemp-based MBCs and identified drying and incubation as the most impactful stages due to high electricity consumption^34^. Similarly, Carcassi et al. emphasised the need to transition to renewable energy sources^60^; however, this shift is often outside the manufacturer’s control and should be pursued as a broader societal goal. In the interim, process modifications can be implemented to reduce energy demand and mitigate environmental impacts. Such modifications could include optimising fungal strain for a specific substrate to reduce incubation time, optimising fungal strain for lower temperature growth to reduce incubation temperature, and as well as alternatives to autoclave sterilisation^72,73^. To improve the A1 stage, reusable moulds with should be used and waste minimised, particularly from disposable materials such as plastic autoclave bags. Regarding A3, alternatives to autoclave-based sterilisation, such as alkali baths or steam pasteurisation, could potentially reduce both the energy demands associated with autoclaving and the reliance on single-use autoclavable bags^72,73^. However, sterilisation remains the most effective method for ensuring successful fungal colonisation^74^. In this study, where a primary objective was to evaluate the feasibility of OSB-MBC growth and production, autoclave sterilisation of the substrate was deemed essential to achieve reliable and reproducible results.

Electricity accounted for 78.2% of the total GWP when the OSB was sourced on-site, again highlighting the importance of reducing energy consumption during production. The sustainability of MBC development is also highly influenced by the energy grid mix; regions with a higher share of renewable energy will significantly enhance the environmental benefits of MBCs compared to those relying on fossil fuels. Previous studies identified that electricity input is important in determining impact variability^58^, however, this may be subject to change due to changes in the production process during scale-up. A carefully managed scale-up strategy emphasising local sourcing, renewable energy use, and efficient waste management will be important for maintaining environmental benefits.

Considering the alternative end-of-life scenarios for waste OSB, the reported C3 (waste processing) contribution to the total GWP calculated in an LCA of OSB (for 1 kg of OSB, data obtained from EPD)^75^, is 1.59 kg CO$_2$ eq. This is approximately 16 times greater than the total GWP calculated for the MBC production in this study. This significant difference underscores the importance of evaluating end-of-life uses for engineered wood waste, with MBC development presenting a more sustainable alternative.

Future research should also consider the practical aspects of construction and demolition waste management required to supply suitable feedstocks for OSB-MBC production. Effective valorisation of engineered wood waste depends on systems capable of collecting, sorting, and concentrating wood fractions from mixed waste streams. Recycling of construction and demolition wood waste typically requires source separation, dedicated collection streams, and material recovery facilities where wood products are sorted and processed prior to reuse or recycling^76^. However, current systems often treat collection, sorting, and recycling stages separately, and integrated frameworks for systematically recovering specific engineered wood products remain limited^77^. Improving pre-demolition audits, on-site sorting, and specialised recycling infrastructure could therefore help concentrate OSB off-cuts and panel waste into relatively homogeneous streams suitable for biological processing. In this study, the OSB originated from clean construction off-cuts; however, post-demolition OSB waste may contain contaminants such as paints, coatings, or fasteners that could influence fungal colonisation or require additional pre-processing. Further research using demolition-derived OSB would therefore help assess the applicability of this approach to more heterogeneous real-world waste streams. In addition to post-consumer waste, sourcing from concentrated streams such as manufacturing off-cuts from OSB suppliers or construction fabrication waste may represent a practical near-term pathway, as these streams are typically cleaner and less contaminated than mixed demolition waste. In addition to OSB waste sourcing logistics, consideration of broader bottlenecks in MBC production is also relevant. The biological growth stage required for fungal colonisation typically takes several days and therefore represents a constraint for large-scale production compared with conventional insulation manufacturing processes. Previous studies have identified incubation time, environmental control requirements, and the need for controlled processing conditions as key factors influencing the scalability of MBC production^78,79^. Developing such supply pathways would support the scaling of OSB-based mycelium composites and contribute to circular strategies for managing engineered wood waste in the construction sector^77^.

While this study focused on the thermal performance and environmental impacts of OSB-based MBCs, additional material properties are important when considering potential applications in the built environment. For insulation materials, performance characteristics such as fire resistance, mechanical strength, and moisture behaviour, and long-term durability are also critical^80,81^. Previous research on mycelium-based composites has reported favourable properties in several of these areas, including inherent fire resistance, moderate compressive strength comparable to other lightweight bio-based insulation materials, and hygroscopic behaviour resulting from the porous lignocellulosic structure^32,36,82^. However, these properties can vary substantially depending on substrate type, particle size, density, and fungal species^27^. Further characterisation of OSB-based MBCs, including mechanical testing, hygrothermal behaviour, and durability assessment, would therefore help to better evaluate their suitability for specific building applications and enable more direct comparison with established insulation materials such as mineral wool or expanded polystyrene. Considering long term stability under service conditions, although mycelium based composites are biodegradable under appropriate environmental conditions, biodegradation generally requires sustained microbial activity, favourable moisture levels, and direct exposure to soil or compost environments^29,32^. In building envelope applications where insulation materials are protected within wall or roof assemblies and maintained under relatively dry conditions, these conditions are less likely to occur^80^. Nevertheless, further research examining moisture durability, biological stability, and the potential use of protective or water-repellent treatments, as well as their implications for environmental impacts, would help clarify the long term performance of OSB-MBCs in construction applications.

In addition to service life durability, the end-of-life behaviour of OSB-based MBCs warrants consideration. While mycelium composites are often regarded as biodegradable due to their lignocellulosic composition^29,32,83^, empirical evidence remains limited^84^. The use of OSB introduces synthetic adhesives, such as polymeric diphenylmethane diisocyanate (pMDI) or phenol-formaldehyde resins, which may persist during biological degradation. Consequently, the biodegradability of OSB-MBCs may differ from that of composites produced from untreated agricultural residues, and further research is needed to assess their end-of-life behaviour. Nevertheless, using OSB waste as a substrate represents a form of waste valorisation that extends the functional lifetime of the material compared with immediate disposal.

The findings establish waste OSB as a viable substrate for sustainable MBC production, with low thermal conductivity and potential as an insulation material. Future research should focus on scaling up production, assessing the durability and hygroscopic properties of OSB-MBCs for applications in the built environment, and evaluating their end-of-life pathways. Additionally, investigating the mechanisms of fungal digestion would yield insights into how mycelium processes adhesives and chemically modifies engineered wood, which is useful for the wider field of waste management and bioremediation. This study underscores the broader potential of fungi to tackle complex waste streams, expanding the scope of substrates for MBC development beyond agricultural residues. Exploring regional and locally available substrates could enhance substrate diversity, resulting in a wider range of applications and material properties. This approach could further advance MBCs as a component of sustainable materials innovation, addressing the need for waste valorisation and for sustainable construction materials.

## Conclusions

The goals of the study were to determine if OSB can be valorised to act as a substrate for forming mycelium-based composites, to determine if OSB-MBCs are effective insulation materials, and to gain insight from a lab-scale LCA of OSB-MBCs. The key conclusions from the study are:

- Fungi (*Trametes versicolor*) are capable of colonising and binding engineered, adhesive-containing wood waste to form mycelium-based composites
- OSB-based MBCs are effective insulation materials with thermal conductivity $\lambda = 0.0415 \pm 0.001$ W/m$\cdot$K
- The prospective LCA indicates that OSB-MBCs could provide a sustainable alternative to conventional insulation materials while offering a beneficial end-of-life pathway for OSB waste, with lower GWP than EPS, XPS, rockwool, and aerogel. The drying stage was the main contributor to environmental impact, and while local sourcing of OSB waste is preferable, transport had only a limited effect on overall GWP.

## Data Availability

The datasets used and/or analysed during the current study are available from the corresponding author on reasonable request.
